# Walk the line: a systemic perspective on stress experienced by emergency medical personnel by comparing military and civilian prehospital settings

**DOI:** 10.3389/fpubh.2023.1136090

**Published:** 2023-06-27

**Authors:** Martine Van Puyvelde, Jolien Van Herck, Jeroen Van den Bossche, Frederic Goethals, Daisy Gijbels, Frederic Detaille, Nathalie Pattyn

**Affiliations:** ^1^Vital Signs and PERformance Monitoring (VIPER) Research Unit, LIFE Department, Royal Military Academy, Brussels, Belgium; ^2^Brain, Body and Cognition, Department of Psychology, Faculty of Psychology and Educational Sciences, Vrije Universiteit Brussel, Brussels, Belgium; ^3^Clinical and Lifespan Psychology, Department of Psychology, Faculty of Psychology and Educational Sciences, Vrije Universiteit Brussel, Brussels, Belgium; ^4^Faculty of Science, School of Natural Sciences and Psychology, Liverpool John Moores University, Liverpool, United Kingdom; ^5^Comd Centre for Mental Health of the Military Hospital Queen Astrid, Brussels, Belgium; ^6^MFYS-BLITS, Human Physiology Department, Vrije Universiteit Brussel, Brussels, Belgium; ^7^Center for Advanced Research in Sleep Medicine, Hôpital du Sacré-Coeur de Montréal, CIUSSS NÎM, Montreal, QC, Canada

**Keywords:** emergency medicine (EM), stress exposure, stress coping, coping strategies, moral injury, SHEL investigation model, mixed method analysis, military vs. civilian

## Abstract

**Introduction:**

Emergency Medicine (EM) personnel in both military and civilian prehospital settings are often exposed to stressful and extreme events. Therefore, a cross-pollination between both contexts in terms of coping strategies may generate new information for purposes of training, prevention, and support programs. In the current study, we aimed at comparing both contexts to understand the type of stress events personnel experience; whether experience differs between civilian and military personnel; and how they cope with it.

**Methods:**

We used a mixed method approach, combining the results of a quantitative questionnaire and a thematic analysis of 23 in-depth semi-structured interviews to gain additional qualitative information.

**Results:**

Whereas the questionnaire pointed to a significant preference for task-oriented coping over avoidant and emotion-oriented coping, the interviews offered a more nuanced insight, showing a constant aim to position themselves on a continuum between emotional disconnection from the patient to preserve operationality on the one hand; and remaining enough empathic to preserve humanity on the other hand. We further identified an ambivalent awareness regarding emotions and stress, a vulnerable disbalance between an excessive passion for the job with the sacrifice of own's personal life (for a growing volatile and dangerous working environment) and a lack of recognition from both the patient and organizational environment. The combination of these factors may carry the risk for moral injury and compassion fatigue. Therefore, mutual trust between the organizational level and EM personnel as well as among team members is crucial.

**Discussion:**

The results are discussed from a systemic SHELL perspective, indicating how the specific profile of EM personnel relates to the software, hardware, environmental and liveware components of their professional and private life. Trainings on stress- and risk awareness should be approached both on an individual and systemic level, knowing that there is clearly no “one-size-fits-all” manner.

## Introduction

According to the International Federation for Emergency Medicine (IFEM), emergency medicine (EM) is “a field of practice based on the knowledge and skills required for the prevention, diagnosis and management of acute and urgent aspects of illness and injury affecting patients of all age groups, with a full spectrum of episodic undifferentiated physical and behavioral disorders; it further encompasses an understanding of the development of pre-hospital and in-hospital emergency medical systems and the skills necessary for this development” (1). This definition underlines, on the one hand, the importance of knowledge and skills and, on the other hand, the need for a flexible adaptation and reactivity capacity to the context, being in-hospital or pre-hospital. The European Society for Emergency Medicine (EuSEM) added the importance of environmental stressors that may impact the conditions of emergency care, such as time pressure, the need to manage the environment as well as triage management, and disaster prevention and care (2).

As stated by Totten and Bellou (3), “war has always been a cradle of trauma innovation” (p. 516), suggesting that EM has strong roots in the lessons thought by war. Nevertheless, in the literature, quite some distinctions are made between civilian and military EM [e.g., (4, 5)]. First, there is a difference in medical care, morbidity, and mortality. In military reports (e.g., Operation Enduring Freedom; Operation Iraqi Freedom), the principal death cause was uncontrollable hemorrhage and the leading causes of preventable death were compressible hemorrhage, tension pneumothorax, and airway and ventilatory compromise (5). In civilian reports, ischemic heart disease and cerebrovascular disease remain the global causes of death in industrialized countries [WHO (6, 7)], also when considering COVID-19 numbers [e.g., Europe (8)]. Second, there is a salient difference in the environmental factors. Within a military context, when the scene of medical care is the theater of war, medical equipment is limited, the treatment conditions are often hostile, and the evacuations are more complex due to tactical risks and longer distances, and thus stretched time periods that need to be tided over. Within a civilian pre-hospital context in so-called first-world countries, hostility is more seldom, the medical equipment is standardized, ambulances are provided, and transport division is generally within foreseen distances (5). To prepare for the specific conditions in the military context, a specialized approach for military medical care, called Tactical Combat Casualty Care (TCCC), has been elaborated about two decades ago (4). TCCC emphasizes that working in war conditions requires well-thought tactical decisions that vary with each specific field circumstance (4, 5, 9). TCCC comprises three phases, i.e., Care under Fire (creating safety for both the casualty and care provider), Tactical Field Care (medical care without standardized medical equipment), and Casualty Evacuation Care (safe evacuation with long time periods). Care under Fire or working in the hot zone is a manner of taking care that is mostly absent in the common civilian EM training (10, 11).

Although the working conditions and injuries may thus differ a great deal between military and civilian contexts, EM personnel in both are exposed to stressful and extreme events. They both operate under high workloads, being regularly confronted with critical incidents (e.g., death, serious injury, violence, and fire) (12, 13). This may menace the success of their commonly used coping strategies (14). When coping fails, these incidents are found to be predictive of posttraumatic stress symptoms (PTSSs) or an acute stress disorder (15, 16). The latter is characterized by transient periods of hyperarousal (e.g., hyper-attention, anger, and irritability), mood changes, intrusive thoughts, or nightmares and may—if chronic—result in a post-traumatic stress disorder (PTSD) [e.g., (12–14, 17–19)]. A PTSD diagnosis (as defined by the DSM-V) requires a direct or repeated indirect exposure to trauma that results in the development of symptoms, for at least 1 month, from each of the following criterion domains, i.e., intrusive memories, avoidance, negative alterations in cognitions and mood, and alterations in arousal and reactivity. According to several recent studies [e.g., (12, 20–23)], PTSSs, depression, anxiety, and PTSD prevalences are dramatically increasing among EM personnel, leading to an alarming rise in personnel loss due to burnout, drop-out, or suicide [e.g., (24–29)].

One of the main mitigators between stress exposure and the development of PTSSs is a well-functioning and resilient stress-coping system (30). According to Lazarus and Folkman (31), coping refers to ‘the constantly changing cognitive and behavioral efforts to manage specific external and/or internal demands that are appraised as taxing or exceeding the resources of the person' (p. 179). Initially, Lazarus and Folkman (31) described two main coping styles, i.e., emotionally oriented (by regulating the emotional reaction to the stress source through avoidance or taking distance) and problem-oriented (by managing, changing, and trying to solve the problem) coping. Among civilian medical personnel, desensitization and task-oriented problem-focused coping (30, 32–34) along with (black) humor (32, 35, 36) have been regularly reported. Similarly, in military personnel, in a study by Morgan et al. (37), problem-solving is the most reported type of coping, followed by talking with a friend or engaging in a distracting (sport) activity (37). According to several authors, the main common goal of using task-oriented coping over emotional-oriented mechanisms is to emotionally disconnect from the patient and its context (33, 34, 38). However, when this task-oriented approach tips the scale to a general emotional disconnection in the individual, persons with PTSSs may risk not seeking support and/or falling back on macho-culture behavior (33, 39–42) or a “big boys don't cry” attitude (33).

Currently, there is a growing consensus that coping is context-dependent (43)—which may imply that coping might vary over different medical contexts, as well as between a military vs. civilian context. However, research that compares the use of coping strategies over different training and operational contexts is rather rare and remains too often restricted to either a civilian or military context. As a consequence, a lack of cross-pollination between both contexts might narrow the information requisite for generating improvements in training, prevention, and support programs (44, 45). Moreover, the majority of these studies are designed from an intra-psychological perspective (e.g., focusing on the PTSSs, burnout symptoms, or coping) without mapping the professional and personal wider system. Hence, in the current study, we aimed at studying stress-coping strategies of well-functioning and mentally healthy persons working in three different prehospital EM care contexts (one military, one civilian, and one mixed miliary-civilian setting, see Method section), considering the interviewees' perspective on both their professional and personal context. Our main research questions were how EM personnel in each of the three settings experience exposure to extreme and stressful events, whether these events have an impact on them, how they cope with it, whether they changed their coping over the years due to experience or age, and how their experiences are framed within their wider personal and professional context. To answer these questions, we applied a mixed method approach, using the Coping Inventory for Stressful Situations [Dutch version, CISS-NL (46)] and conducting in-depth semi-structured interviews.

## Methods

### Participants

The participants were a unique target group of EM professionals without any psychological antecedents nor long-duration sick leave, recruited in three different settings. The first setting considered prehospital EM personnel of a military hospital (MH) in an urban environment; the second setting considered prehospital EM personnel of the Special Operations Surgical Team (SOST), which is a special military unit of medical doctors, nurses, and medical technicians than can be deployed in conflict theaters with little to no medical capacity present but who can be operational in civilian hospitals in between deployments as well; and the third setting considered prehospital EM personnel of a large urban civilian hospital (CH) in the capital city. Twenty-three persons out of 58 contacted persons agreed to participate (see Table 1). The interviews took place a couple of years after the terrorist attacks of March 2016 in Brussels, during which several participants from both the military and civilian settings had been involved as first responders. This makes the current data set unique, as this occurrence was a conflict-type situation (mass casualty event, potential risk to the medical personnel from a second hit) in a civilian setting. The protocol for this study was approved by the ethical committee of the Brugmann University Hospital. All the interviewees signed informed consent before participation. Participation was on a completely voluntary basis with interviewees made aware that they could withdraw at any point.

**Table 1:** Description participants.

**Setting**	***n***	**Mean age in years, *M (SD)* [range]**	**Years of service, *M (SD)* [range]**	**Sex**	**Functions**
Military Hospital (MH)	8	45.6 (9.7) [36–58]	16.6 (16.3) [3–30]	2F/6M	12 Nurses
Special Operations Surgical Team (SOST)	8	44.9 (9.6) [29–54]	17.1 (12.1) [3–33]	8M/0F	9 Medical doctors
Civilian Hospital (CH)	7	40.7 (6.2) [32—49]	13.3 (4.2) [7–20]	5M/2F	2 Medical technicians

### Procedure and design

By selecting the three settings (SOST, MH, and CH), we aimed at developing a “semi cross-over design.” “Cross-over” since it provided information to compare military and civilian personnel, both operating in a civilian context; “semi” since—inherent to the job requirements—only the military personnel and not the civilian personnel could operate on military terrain.

Each interviewee completed the questionnaire under the supervision and was then interviewed. Each interview lasted between 60 and 90 min and was completed in person, recorded, and transcribed. The interview questions consisted of four main sections: 1/demographics, career, training, and mission specifics, 2/extreme stress exposure: what do EM personnel perceive as extreme and what is the eventual impact of it with as leading questions “do you think that medical emergency personnel are at risk for extreme stress exposure?” and “have extreme stress events an impact on you?”, 3/coping strategies and experience, which explored how the participant coped with extreme stress exposure and whether experience played a role in coping success, and, 4/job motivation, to explore the core motivation and worthwhile professional memories of the participant. These questions were constructed through an iterative explorative discussion by operational EM experts and trauma psychologists of the Center for Mental Health in the Military Hospital in Brussels. The questions were tested out for feasibility (i.e., open questions that provide a balance between in-depth opportunities without being confronting).

### Materials

In the quantitative part, we used the Coping Inventory for Stressful Situations (CISS-NL, de Ridder & van Heck, 2004 and Rolland, 1998) [accepted versions of the original Canadian version (47, 48)]. The questionnaire contains 48 items with 5-point response scales (“not at all” to “very much”) that cover three coping styles, i.e., the emotion-oriented, task-oriented, and avoidant coping styles. There are norm tables provided over seven categories (from “very high” to “very low”) that include gender differentiation. Statistical analyses were performed using SPSS, version 27.

In the qualitative part, semi-structured in-depth interviews were conducted, transcribed, coded, and analyzed, using NVivo (© QSR International, release 1.6).

### Qualitative analysis

The analyses were conducted using the inductive thematic analysis approach of Braun and Clarke (49), which we expanded with a triangulation method (50) to ensure the truth value. The inductive thematic analysis is a non-linear recursive method based on six phases of analysis that need to be flexibly applied, demanding a constant back-and-forth movement through data. It is an inductive bottom-up approach that takes the data as a starting point. In accordance with Braun and Clarke (49), in the first phase, we familiarized ourselves with the data by transcribing, reading, listening, and taking notes. In phase two, we generated more than 400 initial codes in a bottom-up manner reflecting the content themes in the data. On the one hand, we systematically coded relevant features of the data and, on the other hand, collected further data that corresponded to each code. Afterward, in the third phase, the potential overarching themes were identified with the goal to organize the data in clusters. In phase four, the themes were reviewed by checking cohesion among the theme, coded parts, and the entire data set. Then, in the fifth phase, the codes were defined, specified, and labeled to prepare the final sixth phase or results section that was finalized in a model.

To ensure the truth value (51) or credibility (52), we applied the triangulation method (50) to establish “how confident the researcher is with the truth of the findings based on the research design, informants, and context.” By applying the triangulation method, we aimed at increasing the validity of the results by a crosscheck over different sources such as methods, literature, concepts, and researchers. In correspondence with Carter et al. (50), two researchers participated in the analysis process in the first bottom-up stage and a third researcher with extensive experience as an EM medical doctor was added in stage three to review and reflect on the resulting themes, categories, and interpretations. According to Sandelowski (53), a qualitative study is credible when it presents such accurate descriptions or interpretations of human experiences that individuals who shared similar experiences would immediately recognize them.

### Statistical analyses

To compare the CISS dimensions over the three settings, a 3 × 3 [coping (TASK, EMOTION, AVOIDANT) × setting (MH, SOST, CH)] mixed ANOVA; a 3 × 3 [coping (TASK, EMOTION, AVOIDANT) × years of service (<9, 9–20, >20)] mixed ANOVA; and a 3 × 2 [coping (TASK, EMOTION, AVOIDANT) × gender (man, woman)] mixed ANOVA were calculated with the coping style as within-subjects, the setting/years of service/gender as between-subjects factors, and the scores as dependent variables. The usual cutoffs to interpret effect sizes were used for small, medium, and large effect sizes (54). Additional pairwise comparisons were conducted where applicable, with the critical *p*-value for significance adjusted with the Bonferroni correction.

### Anonymity in reporting results

For reasons of anonymity, we indicated neither the participant number nor the specialization (medical doctor, nurse, or technician) in the text citations in the Results section. We only mentioned the EM setting (SOST, MH, or CH). The text citations were evenly distributed among the different participants.

### Truth value

The third researcher confirmed the validity of the themes identified through analysis as well as the final model presented at the end of the results section that interconnected these themes.

## Results

In this section, we will present the main themes generated by the inductive bottom-up analysis. Additionally, to differentiate between the three settings—per discussed phenomenon—we indicated by how many interviewees (%) a respective topic was discussed.

### Stress exposure, stress perception, and stress resistance

All the interviewees described several examples of what (extreme) stress means to them, depicting mass events, accidents, events with serious injuries and/or vulnerable victims (e.g., children), extremely high workload, and patient flux. Being exposed to danger was often mentioned (100% SOST, 75% MH, and 57% CH) but there was a difference in the type of danger between the three settings. In SOST personnel, the danger was war-related, and people felt mentally prepared for it, whereas, in CH and MH personnel, the danger was experienced as an unpredictive and individually directed, personal threat for which they did not feel prepared (see Supplementary Table 1).

In a civilian setting, the most mentioned impactful event (100% MH, 57.5% CH, and 50% SOST) was the injury or death of a(n) (unborn) child (pregnant women included, considered as the risk to lose two persons): “*you feel there is a silence afterwards, but afterwards we talk about it in the team*” (MH). The death of a child is experienced as unjust, and therefore sometimes as a personal unacceptable failure. It raises associations with their own children, making it difficult or sometimes impossible to keep an emotional distance from the patient. For the same reasons, treating a familiar person was said to be very stressful. Furthermore, the unbearable grief of the family was mentioned repeatedly as potentially traumatic. In a military setting, the 2016 terrorist attacks in Brussels were the most impactful:

“*The most impactful was March 22. It was the worst situation we had, for me, because it was at home, with us… if you're on deployment, you get on the plane and you return to Belgium and in fact you can switch your button … when you're returning you're resetting yourself… but on March 22 you didn't have a button to switch because suddenly they were all standing here … it had more impact than all the missions together, March 22, I find*.” (SOST).

Acute physiological stress responses, in terms of fight, flight, or freeze responses were reported recurrently. Some personnel (50% SOST, 25% MH, and 42.9% CH) reported the positive impact of stress that it makes you do things that you would never think you could: “…* he has started to shout and yell and 10 min later he had arranged everything… he really rose above himself* ” (MH). Physiological stress was also said to be an ideal observation tool to be aware of one's inner state: “*I know when I am stressed, my voice starts to change, if my heart rate starts to increase, you know immediately…*” (CH). On the other hand, stress could also carry the risk to freeze and block further adequate operational performance:

“…* it really shocked me, it is not something that stayed, but at that point it frightened me, because it was the first time that I was not capable to come into action. And thus, the nurse, who was with me, she kept on prodding me and talking to me, to bring me back into the moment. I told her afterwards, it was such a luck, she did that because I was useless at that point*.” (CH).

Regarding chronic stress exposure, the responses were more complex in a way that the explicit responses to the questions “do you think that medical emergency personnel are at risk for extreme stress exposure?” and “do extreme stress events have an impact on you?” did not always correspond with what interviewees described throughout the interview implicitly (without that the interviewer asked for it) using examples that contained several illustrations of PTSSs' (changes in) behavior, cognitions, and mood that were given throughout the interview. Table 2 overviews, for each individual, how they responded to questions 1 and 2, what setting they were part of, what PTSSs they eventually experienced, and to what extent they were aware of that. Furthermore, it is notable that the experience of chronic stress is variable over the years and interpreted differently between individuals. For instance, four out of 23 persons (2 SOST and 2 MH) did not agree that EM personnel was at risk for extreme stress exposure. They argued that the feeling to be at risk for extreme stress exposure is dependent on what you as a person perceive and experience as extreme, assuming that a certain level of stress resistance is necessary or needs to be acquired over the years: “*if you can't give it a place in your mind, you will not be able to persevere*” (MH). Ten interviewees (7 SOST, 2 CH, and 1 MH) argued that stress events did not impact them (anymore): “*it doesn't touch me … I think I have put barriers: for me, whether there is one dead person or there are ten, it will not change anything, I just want to do my work well and I am not touched anymore with regard to humans*” (MH). The majority of interviewees (75% SOST, 62.5% MH, and 87.5% CH) testified that they learned through the years to better manage stress and that the impact of certain interventions decreased with experience. “*I remember, the first interventions …. pfff…. I returned home, I was empty, incapable to say a word … but you need to find a solution, euhm, because if it doesn't go well, you need to stop, you know*” (SOST). However, one person added that age and experience can have a reversed effect as well, in the sense that you think “*my, goodness, I have survived all that, I hope it will go well once again*” (SOST).

**Table 2:** Stress awareness.

				**Setting**	**PTSS criteria and degree of awareness**	
Are medical emergency personnel at risk for extreme stress exposure?	No	Do extreme stress events have an impact on you?	Yes, but dependent from situation. Rather on others.	MH	/	/
			In the past, but dependent of the situation	MH	/	/
				SOST	B	3
					E	3
			In the past. Also seen in others.	SOST	E	2
	Yes		In the past, but dependent of the situation. Also seen in others.	SOST	/	
				SOST	C	0
					D	1
				SOST	B	0
				SOST	/	
				SOST	B	0
					E	3
				CH	E	1
			Yes	CH	C	3
					E	2
				CH	B	3
					C	2
					D	3
					E	3
				CH	C	2
					D	2
					E	3
			Yes, but dependent from the situation.	MH	D	3
					E	2
				MH	B	1
					E	1
				MH	B	3
					C	1
					D	3
				MH	/	/
				SOST	B	0
					D	3
					E	2
				CH	E	3
				CH	B	3
					D	3
			Not on me but on others.	CH	B	0
					C	3
					E	2
			No	MH	B	3
					C	0
					D	0
					E	0

This table displays a representation of how the interviewees experienced stress explicitly and implicitly. Explicitly: the responses refer to the questions whether the interviewees thought that emergency personnel is at risk for extreme stress exposure (columns one and two) and whether they thought stress events may impact them (columns three and four). Implicitly: whether they mentioned examples of post-traumatic stress symptoms (PTSSs) throughout the interview without it being explicitly asked by the interviewer and if so what type of PTSSs (column 6). We also added for each interviewee their setting (column 5 and a degree of awareness (column 7), i.e., the extent that the interviewee assigned certain (changes in) behavior to stress or not). The table must be read in a vertical and horizontal manner. Vertically, as explained earlier. Horizontally, in that, one can read from the left to the right, for each individual, how they responded to questions 1 and 2, what setting they were part of, what PTSSs they eventually experienced, and to what extent they were aware of that.

PTSS criteria are “B” intrusion symptoms; “C” avoidance; “D” negative alterations in cognition and mood; and “E” alterations in arousal and reactivity. The degree of awareness refers to “0” no awareness; “1” recognized in colleagues; “2” someone made them aware of their described behavior; and “3” self-aware.

Independent of how interviewees perceived stress exposure, it was regularly mentioned that every person has a limit in coping with stress and only three interviewees agreed that stressful events have an impact without stating conditions. They all reported PTSSs and reflected on the potential risk of stress exposure in their job:

“*These are memories, I know that the day I will have a situation, not necessarily malicious, a knife attack, bullet wound, maybe just a difficult situation with a patient or family, it will certainly come back to me again…*” (CH).

### Other stress sources that may cumulate with event-evoked stress

#### The specific environment: to work with what you have

Emergency medicine field conditions are seldom optimal (e.g., lack of light, victims found in small spaces, car accidents, weather, and war scene). Moreover, heat, noise, seasickness, and unhealthy or irregular food intake may impact the mental and/or physical condition of the personnel (87.5% of SOST) (see also Supplementary Table 2).

#### The relationship between the organization and em personnel: a mutual cycle of trust

Limited resources are an important source of stress (62.5% MH, 75% SOST, 50% CH). A lack of personnel was the main recurring complaint, followed by the lack of budget to follow necessary training and a lack of logistic support for military personnel when on deployment (75% SOST). Finally, SOST personnel often complained about a lack of tactical preparation (e.g., shooting and survival). When resources are limited, this may feed physical fatigue, burn-out, and above all, a loss of trust, creating a sense of not feeling recognized or disrespectfully treated. This negative cycle was said to carry the risk for a decrease in motivation and commitment in both a military and civilian setting: “… *that's why people leave*” (SOST), “*a lack of recognition, … that's a gigantic source of stress in our profession*” (CH) (see also Supplementary Table 2).

#### The impact of the job on family and vice versa

Most of the interviewees mentioned the big impact the job has on their family life (100% SOST, 75% MH, and 57% CH) and vice versa (25% SOST, 37.5% MH, and 28.5% CH). The life partner must be strong, flexible, monitoring, and supporting and should accept the sacrifices made by the EM personnel (see also Supplementary Table 2).

### Stress coping strategies and their potential drawback

#### Quantitative analysis

##### The Coping Inventory for Stressful Situations (CISS)

Based on the CISS, the participants reported significantly more task-oriented coping than emotion-oriented and avoidant coping (*F* = 86.31, ηp^2^ = 0.797, *p* < 0.001, Bonferroni corrected) and more avoidant than emotion-oriented coping (Bonferroni corrected, *p* < 0.001). There were no effects of years of service (*p* = 0.943), setting (SOST, CH, MH) (*p* = 0.445), or sex (*p* = 0.869). Comparisons with CISS normative tables showed that the task-oriented and avoidant coping style was within norms in both male and female participants and the emotion-oriented coping was below average in both male and female participants. Hence, in the current population, emotion-oriented coping was underrepresented. Although no significant effects of setting were found based on ANOVA, when comparing with normative tables, an underrepresentation was most present in the SOST-male category and persons with lower years of service (see Table 3).

**Table 3:** Coping styles per sex, setting and years of service, and total.

	**Sex, M (SD)**		**Setting, M (SD)**		**Years of service, M (SD)**	
Avoidant coping	M	41.47 (11.50)	SOST	40.25 (9.44)	≤9	39.29 (11.04)
	F	45.50 (10.28)	MH	39.88 (13.021)	10–19	43.63 (12.40)
			CH	47.00 (10.83)	≥20	43.25 (11.07)
	Total 42.17 (11.18)					
Emotion-oriented coping	M	31.84 (7.86)	SOST	30.75 (7.34)	≤9	28.57 (9.88)
	F	36.00 (9.20)	MH	32.25 (9.85)	10–19	32.63 (4.31)
			CH	31.57 (6.78)	≥20	36 (8.47)
	Total 32.57 (8.04)					
Task-oriented coping	M	62.00 (5.94)	SOST	59.38 (7.72)	≤9	57.43 (4.43)
	F	63.25 (11.03)	MH	62.13 (6.15)	10–19	63.00 (7.98)
			CH	65.57 (5.47)	≥20	65.63 (5.13)
	Total 62.22 (6.76)					
	*F =* 0.140, η_p_^2^ = 0.007,	*F =* 0.140, η_p_^2^ = 0.007,	*F =* 0.951, η_p_^2^ = 0.087,			
	*p* = 0.869, power = 0.070	*p* = 0.869, power = 0.273	*p* = 0.445, power = 0.086			

#### Qualitative analysis

##### The balance between emotional distance and empathy

One of the most discussed topics throughout the interviews was the search for balance between being sufficiently emotionally distant toward the victim and their family and having enough empathy for them: “*And I knew I had to find the balance in between putting barriers to protect myself but still permitting to take care of the patient in his globality*” (CH). Inherent to an EM the setting is a short-in-duration contact with the patient (contrary to an intensive care unit where a doctor–patient contact is long and intensive). This fleetingness facilitates taking distance from the patient and helps to avoid feeling too emotionally engaged. The disadvantage, however, is a potential lack of information about the patient's outcome, and thus about the outcome of one's work. A lot of interviewees testified to the urge to inquire in the hospital about the patient's state. Moreover, there is no time or space for patients to express their gratitude: “*We are not in the setting where you receive chocolate boxes from the family*.” During military missions, very similar experiences were reported. The lack of close and sustained contact with the patient, i.e., just the time to stabilize the patient and prepare them for transport, created enough distance to avoid emotional engagement (see also Supplementary Table 3).

##### Avoidant coping and the risk for escapism

We encountered examples of avoidant coping in 100% of the SOST, 87.5% of the MH, and 85% of the CH personnel. The most common avoidant coping mechanism was to switch off a button and to emotionally disconnect from the source of stress or danger. In addition, feelings of habituation and getting indifferent were mentioned (which may only be realized retrospectively): “*on beforehand, you realize, but, euhm, you don't care… euhm… you are so far in the trauma or in your capacity to survive that you don't care anymore. And euhm, so afterwards, I was another person when I returned…*” (SOST). Becoming indifferent, alcohol use, and suicide were mentioned as examples of escapism and also relaxing activities such as sports or sleeping (see also Supplementary Table 3).

##### Macho culture or john wayne syndrome

A variation of avoidant coping may be found in the so-called John Wayne syndrome or macho culture. Fifteen persons (50% SOST, 62.5% MH, and 86% CH) confirmed that a macho culture did exist and 12 persons (37.5% SOST, 50% MH, and 71.5% CH) explained that a macho culture is an existing part of what you could define as “a flashing light culture” (i.e., enjoying the sensational stories of an EM service) and that it is more present in the older colleagues than in the younger generation: “*You know with whom you can talk … I know that some people can't because they think they will be judged and that you show weakness…*” (CH). Some interviewees agreed that more emotional persons may not be made for the job whereas others argued that a macho mentality may hamper searching for support and talking or showing sincere empathy for the patient and their family (see also Supplementary Table 3).

##### Emotion-oriented coping and the need for reassurance to avoid self-blaming

The most reported way of emotional coping was to talk about an intervention (62.5% SOST, 100% MH, and 85% CH). Within a professional context, informal debriefings are the most common and preferred way (e.g., in the vehicle when returning from an intervention). These moments allow us to evaluate the actions and decisions made and to find reassurance that everyone did the best they could. The latter appeared crucial when dealing with the loss of a patient, that is, to learn to accept that one cannot save everybody (75% MH, 50% SOST, and 85% CH).

Some interviewees preferred not to discuss interventions with colleagues but were happy to listen to others when necessary. Some SOST members mentioned that they would never discuss their missions with non-SOST members due to former experiences in which they were not believed. For similar reasons, about half of the interviewees avoided discussing their work with family or friends since, according to them, the outside world is incapable of understanding their job. They strictly separated their professional and private life (to the point of hiding from family and friends that they experienced the 2016 terrorist attacks in Brussels). Concerning consulting a psychologist, barriers existed. Less than half of the interviewees mentioned having (limited) experience with a psychologist. For about 63%, this experience was positive, 27% neutral, and for one person, it was negative. Finally, humor and staying positive were regularly used coping strategies (75% SOST, 62.5% MH, and 43% CH) (see also Supplementary Table 3).

##### Task-oriented coping and the risk of emotional disconnect

Focusing on the task, the protocol or algorithm facilitates emotional disconnection toward the patient and their family (100% MH, 87.5% SOST, and 57% CH), and following a structured problem-solving approach, guided by protocols, aids to be sure to have done the best they could. Procedures and protocols were said to structure the operational performance, facilitate post-intervention debriefings, and improve situational awareness in mass events (85.5% CH, 62.5% MH, and 37.5% SOST), whereas a checklist is common in single interventions (e.g., ambulance material). Protocols and procedures may have disadvantages as well and are still far from up to date (e.g., the 2016 terrorist attacks in Brussels put to the proof certain protocols). They may overshoot the goal of a particular situation or give a false sense of safety (see Supplementary Table 3).

##### Team investment: another mutual cycle of trust

The emergency units are small, horizontally functioning units. In CH personnel, the medical doctor and nurse work closely together, as a binomial, alternating in taking decisions and relying on one another. Hence, the differentiation between doctors and nurses often fades away. Within a SOST team, no military rank counts when a patient is on the table. Moreover, in SOST members, during deployments, besides working together 24/7, the team also must live and sleep together in a small space, as a family, as they phrase it themselves. Obviously, transparent communication and mutual respect for one's privacy are primordial when working closely in small teams. Mutual respect is the interpersonal glue between members, and any conflict among team members is a huge source of stress and may block proper working conditions (75% MH/SOST and 57 CH). A threat to trust may, therefore, put at risk the entire team functioning whereas positive team dynamics reinforce self-confidence on an individual level. Furthermore, in the military setting, about 50% of the interviewees emphasized the importance to know one another and to dare to speak up (see Supplementary Table 3).

### Training and skill or knowledge decay

We encountered several descriptions of situations that may comprise a risk for skill or knowledge decay (87.5% SOST, 87.5% MH, and 57% CH) (e.g., lack of retraining, the alternation between long-duration deployments and local medical care requiring different approaches, and the combination of medical and managerial/administrative jobs). Nevertheless, explicitly, only six interviewees (2 SOST, 2 MH, and 2 CH) discussed the importance and/or lack of (re)training:

“*I always was afraid of children and childbirths. I have paid for a specific training to learn that by myself and thus now I am comfortable with it. Even in a difficult situation… (describes an intervention with a baby) … other colleagues will certainly have a loss of competence…*” (CH).

### My heart will go on: the psychological profile of EM personnel

Medical personnel goes on, despite the stress they are exposed to, and the sacrifices of their private life because of their passion or almost addiction to the job (“junkie for the job,” 62.5% SOST, 75% MH, and 28.5% CH):

“*I became a junkie… addicted to the way of cooperating, humanity. You are full blown doing something good, you score in a good way, you have a good team, you can count on everybody… It is so intensive that it makes you a junkie, I find*.” (SOST)

They are eager to learn and the job teaches them important life lessons such as putting things into perspective, appreciating the meaning of life, and getting to know other cultures (mainly SOST). Moreover, the desire to help and save people is a strong motivator and EM personnel always search to generate moments of happiness:

“*these two girls, on Monday, they were 10 and 3 years old. We had brought them to the same hospital and one girl had already arrived, lying there. The other was laid next to her older sister, only 10 years, and she takes the hand of her little sister…*” (CH)

Emergency medicine personnel are attracted to challenges and adventure, and sometimes even enjoy being in danger. They prefer variation above routine (>50%) and possess the flexibility and capacity to adapt quickly to acute, unpredictable, and changeable situations. Problem-solving thinking is crucial and the team makes it worthwhile to continue.

### Human factors SHELL as a model for EM operationality

**Figure 1:**
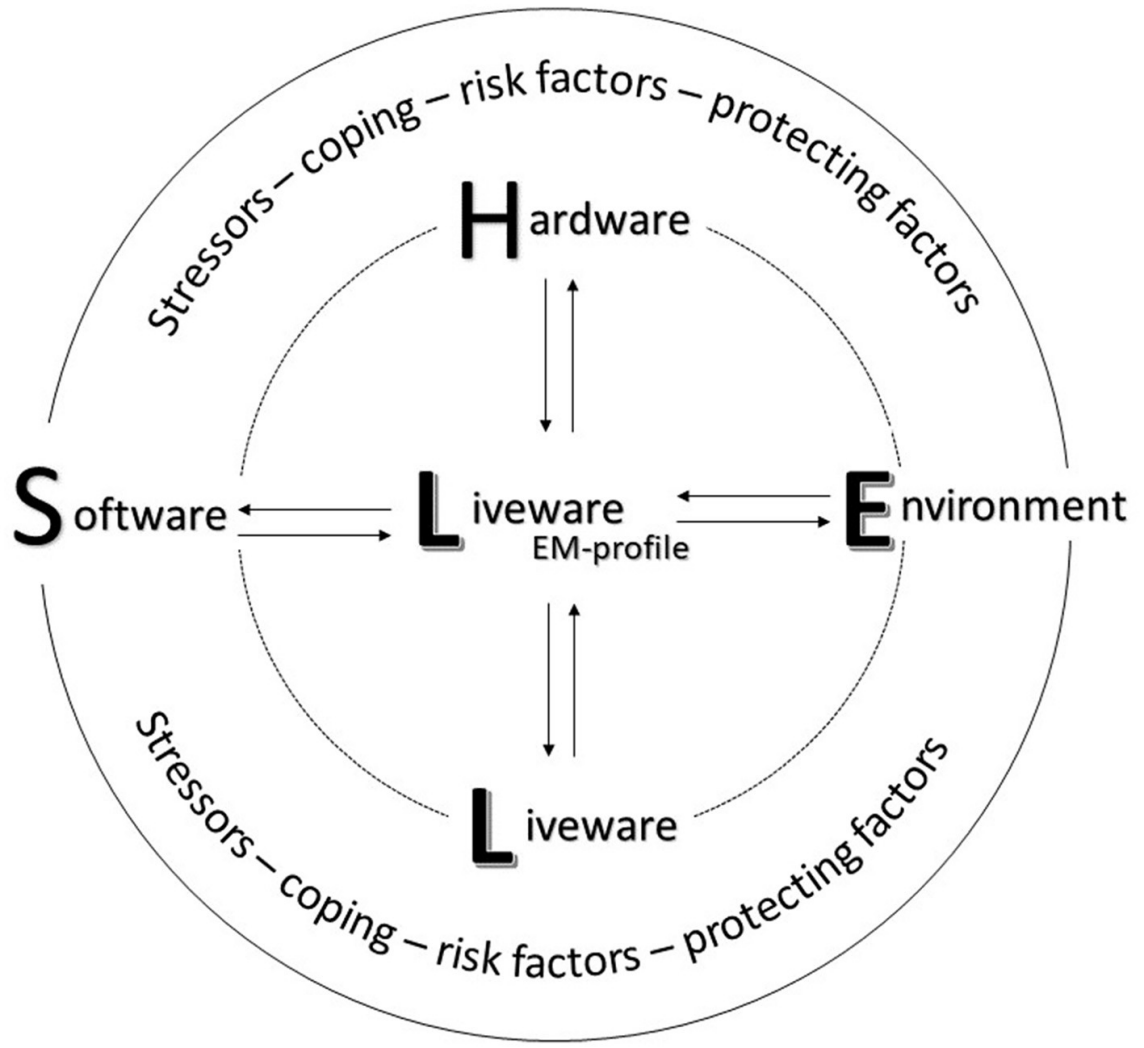
Overview of the model for EM operationality based on the human factors SHELL model, showing the multifactorial personal and professional context of EM personnel.

The obtained categories correspond with the key components of the SHELL model (55, 56). The SHELL model is originated from SHEL by Edwards in 1972 and further developed by Hawkins (56). It is a model of human performance in professional environments that has been developed within the multidisciplinary science domain of Human Factors to optimize efficiency and safety. In the SHELL model, the interrelationships among a working individual (L: liveware or here the EM profile) and the procedures they need to apply (S: software), the tools they need to use (H: hardware), the environment in which they need to function (E: environment), and the personnel they need to work with (L: liveware) are studied (55, 56). All the encountered stressors, coping strategies, risk factors, and protective factors are the expression of an interaction between the EM personnel and one or more SHEL components (Figure 1).

## Discussion

In the current study, we aimed at examining the profile and working environment of EM personnel in both a civilian and military context in order to understand the type of stressful events they are exposed to and how they cope with them, and how their experiences are framed in their wider personal and professional context. We used the CISS questionnaire and conducted semi-structured interviews to gain additional qualitative information. The interviews showed that there was no one-size-fits-all and straightforward answer to our research questions. Instead, the responses of the interviewees showed a strong multifactorial character, showing an interaction between the individual and the different interrelated layers of the operating system in which EM personnel is functioning. In this discussion, we aim to highlight the most important topics by respecting these multifactorial context properties.

The CISS questionnaire showed a significant preference for task-oriented over avoidant and emotion-oriented coping, with the latter being below norms. Similar findings have been reported in previous studies [e.g., (30, 32–34, 37)]. In these studies, this coping preference was explained as a manner to protect oneself against being emotionally overwhelmed by the situation. Using task-oriented coping during crucial events was described by some interviewees as well. In extremis, a focus on the medical task blocked post-retrieval of certain environmental details such as the color of a victim's car or the persons they communicated with (e.g., during the 2016 terrorist attacks in Brussels). This confirms a study by Jonsson and Segesten who described similar testimonies (57). However, task-oriented coping is not a one-size-fits-all framework. The story is more nuanced. From the interviews, it became clear that EM personnel are in a continuous attempt or sometimes even struggle to find an optimal position on a continuum between emotional connection and disconnection. On the one hand, they strive to have enough emotional distance to preserve their operational performance, but on the other hand, they must maintain enough empathy for the patient and their family to remain human; and this search for balance in humanity appears to be an important and very individual pathway to walk. Some interviewees testified how they had to search to become less emotionally engaged over the years in order to be able to perform and not take their interventions homeward, whereas others testified the other way around. They had to be careful not to become indifferent (58).

Hence, the limit between operationality and emotional engagement is a thin line to walk, which might explain certain incongruencies we detected in some testimonies between the explicit opinion about stress exposure and the implicit experiences when describing emergency events (*e.g.*, “…* this was horrible, sick making. Because of its extremity, I guess…*,” *SOST)*. According to Lane (59), such incongruencies are the result of a lack of emotional awareness, due to processing that is present on an implicit level without making a meta-cognitive connection. Hence, in explicit memory, a connection between, or awareness of, the experienced event and related emotional and/or physiological responses is lacking, which may block the building of psychophysiological stress-management strategies (60–64). Several biobehavioral brain studies (59, 62, 65, 66) have indeed shown that a lack of emotional awareness interferes with the capacity to detect, recognize, and interpret (bodily) stress responses, impacting mental health in terms of stress vulnerability and PTSSs (60–64).

In the literature, one of the main protective factors against PTSS remains seeking social support in the form of talking with a friend or colleague, be it formally or informally (33, 37, 39, 57, 59, 67–71). However, both in the literature and in the current interviews, one of the main barriers to seeking support is the fear to be perceived as “weak” and being stigmatized as not being able to perform the job (25, 39, 72–75). This attitude may lead to the idea that extremely stressful and untreated potentially traumatizing events are the norm rather than the exception (41, 57) and, in extremis, could nurture feelings of indifference and compassion fatigue (76–78). This “big boys don't cry” attitude (33) was present in some of the current interviews as well. The only way of consultation that was appreciated by most of the interviewees was informal debriefing such as during a drive back in the vehicle after an intervention, which confirms recent research on firefighters (58). Support from family and friends, on the contrary, was often dismissed. Almost 50% of the interviewees did not talk with their family or friends about their job, being convinced that an outsider would never understand. Nevertheless, almost 80% reported that their job and certainly the fact that the job was not discussed at home had a negative impact on their family life. Some interviewees were divorced and appeared to consider this almost as a normal sacrifice to make, being part of the job, being the rule rather than the exception. Nevertheless, both the current interviews and previous studies have shown that by not sharing their experiences with family and friends, one can be pushed toward an isolated and marginal position in private life (26, 70).

Hence, there is a tendency to master and control when and to whom one will talk. We suggest this is an implicit manner of stress regulation as well, related to the often-mentioned coping strategy to seek reassurance on performance (79). By reviewing every optional solution for an encountered problem during an event, any feelings of self-blame can be ruled out. Self-blame remains persistent among EM personnel (26, 80, 81) and its elimination is shown to be crucial in the prevention of burnout, PTSSs, and extremis suicide [e.g., (26, 57, 80–83)]. The obstinacy of self-blame was shown in a study by Bian et al. (80) in which a 14-week coping training program for civilian and military emergency personnel was tested. The training significantly increased the use of strategies such as problem-solving and help-seeking and decreased the use of phantasy, avoidance, and rationalization. However, self-blaming did not change, signifying that the latter is truly difficult to change and may be persistently inherent. Therefore, according to Jonsson and Segesten (57), trainers should be much more attentive to the major role mechanisms of guilt, shame, and self-blame play in coping and trauma processing. Moreover, a difference should be made between guilt (which is a painful feeling about one's actions) and shame (which is a painful feeling about oneself, not only the actions but the individual's self and identity) (57). This may explain the preference to discuss with experts over non-experts. That is—from this perspective—a professional discussion with a colleague guided by medical expertise will focus on the actions and will therefore, at worst, touch feelings of guilt; whereas a discussion with a non-expert would risk entering the zone of shame.

**Figure 2:**
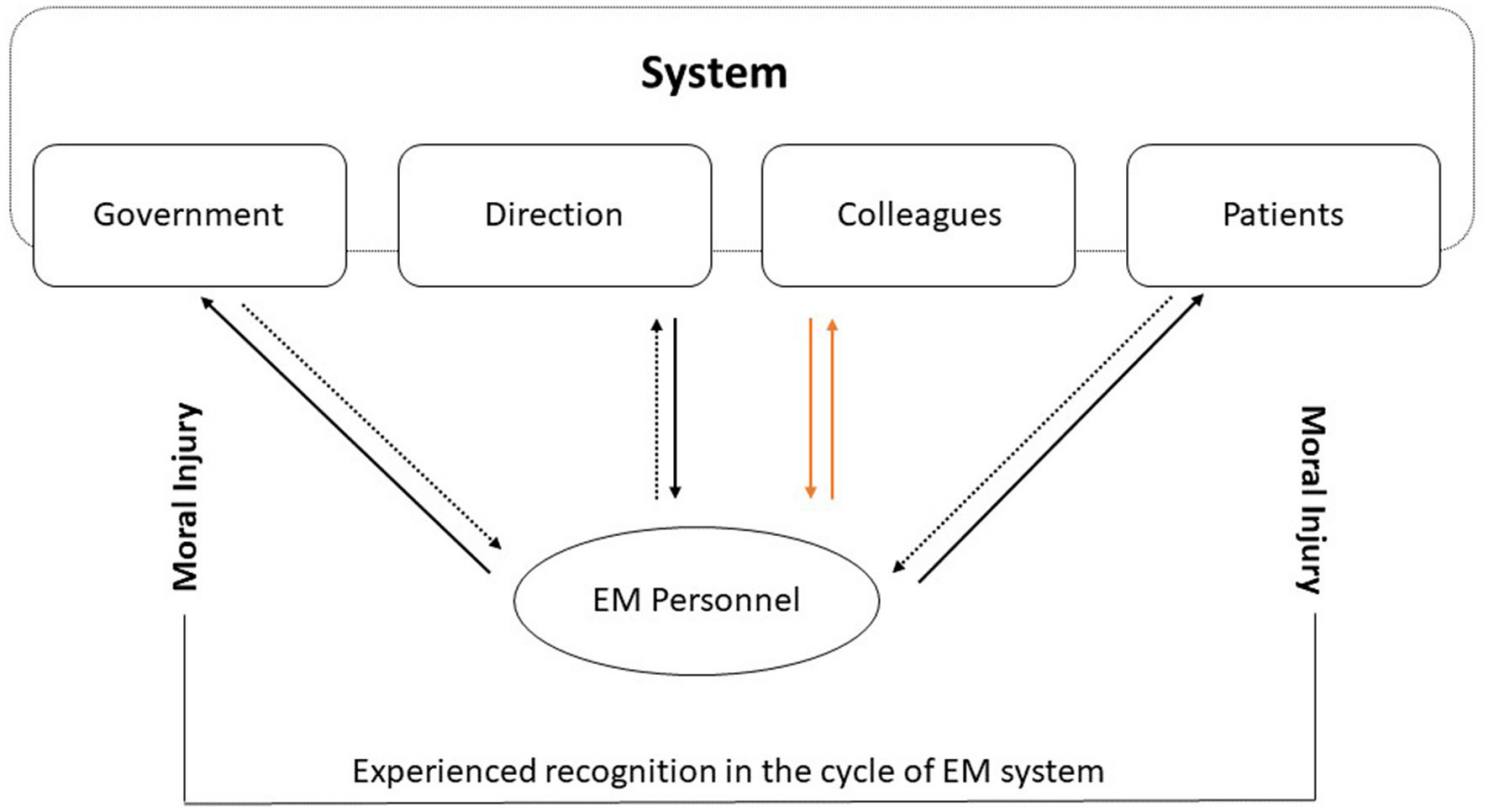
The cycle of risk for moral injury as observed in the current interviews. When mutual recognition between different layers of the system and the individual is lacking, increased pressure in terms of overcompensation may occur among team members. The idea of broken mutual trust among team members is extremely stressful and increases the risk of moral injury.

Although concepts such as self-blame, burnout, and PTSSs are often approached as intrapersonal concepts [e.g., (84, 85)], the interviews showed they are embedded in a multifactorial environment or system. A broader systemic perspective on burnout is offered by the concept of moral injury (86–88). Moral injury is a concept borrowed from the military domain, based on observations during the two World Wars showing a “reluctance to kill” due to ethical and moral beliefs (89). It refers to any blockade that prevents further action when confronted with a situation that transgresses our deeply held moral beliefs such as the impossibility to put a patient's care first (87). Rather than being burnt out, some physicians may feel blocked in their striving to help the patient due to a lack of personnel, technical, and non-technical training and logistic support. When one is fulfilling their job with a passion “as strong as a junkie,” putting the patient first at the expense of one's own personal life quality, he or she needs to be supported and recognized by the broader system. If not, this sense of loyalty risks being exploited and the disillusion can be great (90). Since, in EM settings, recognition will seldom be found in patients due to the very brief patient contact, it should certainly be present at an organizational level. If not, the source of work validation risks to become limited to the team members which may put the team(ness) under pressure (see Figure 2).

Indeed, mutual trust among team members was recurrently mentioned as one of the most crucial components of the interviewees' life, even more in SOST members than non-SOST members. In a military context, the team is the building block of a larger functional unit (91) and interpersonal communication is primordial to optimize operationality and avoid counterproductive conflicts (92). This was omnipresent in the interviews as well. On deployment, the team lives together for months as a small family in rather rustic and sometimes stressful circumstances. They rely on one another for 24/24 h, working long shifts, being confined, and without having any post-shift privacy. Obviously, without optimal communication, these circumstances would quickly become a hotbed of interpersonal conflict (93, 94). Even in the non-SOST members, team spirit was said to be primordial, and eventual team conflicts were for some of the interviewees a greater source of stress than a critical event. EM units are small—even binomial between doctor and nurse in the civilian setting—and have a non-hierarchical horizontal manner of working [e.g., (95, 96)].

The comparison of the three settings and how they can cross-pollinate one another was most visible when discussing the environmental working context of EM personnel. Both the MH and CH interviewees worked in a main city dealing with increased aggression [e.g., recent Belgian (97) and Australian (98) reports on physical and verbal aggression]. These increased violent working conditions are resembling what has been previously defined as a *volatile, uncertain, complex, and ambiguous* (VUCA) environment (99) which is much more familiar territory for SOST operators. Operating in a VUCA environment demands a high situational awareness, the readiness to take risks, and the flexibility to adapt to changing and challenging situations (100). An extreme example in the interviews of the rising VUCA environment was the 2016 terrorist attacks in Brussels. An illustrative example of the unexpectedness or unpredictability of this event is the anecdote of one of the interviewees, who presumed that the radio communicator was making a joke when announcing that they had to leave for the airport for a bomb attack. From one moment to the other, existing protocols were challenged and even found useless. However, SOST personnel reported they were able to rely on certain strategies and care training such as the TCCC training (4, 5). Indeed, in programs such as Tactical Emergency Medical Support (TEMS) (10) and Tactical Emergency Casualty Care (TECC) (101), the TCCC care-under-fire tactics are trained by civilian personnel to learn to work in hot zones to attempt to minimize the therapeutic vacuum or time period during which medical care is blocked by a threat (102). Nevertheless, the psychological impact was mentioned to be great as well in most of the SOST members, who testified that their usual “switch-off-the-button-on-the-plane-when-returning-home” coping strategy did not work here. This was an event that happened here, on their territory, close to their own home, in their own living environment, wherefore it was more difficult to switch off and change the channel.

Finally, although we identified several implicit examples throughout the interviews that carried a clear risk for skill decay, this risk was rarely discussed explicitly. Nevertheless, medical skill decay is a potential risk in military, civil, and academic medical settings. Both in the interviews and in the literature [e.g., (103–107)] (e.g., switch between military and civilian contexts, protracted absence through illness due to burnout, lack of training), we suggest that this bias may be partly cultural. For instance, in the UK, skill decay is acknowledged, and retraining is part of the National Health Service (NHS) accreditation procedure (108) and thus an obvious part of the professional risks to have attention for, which is not the case in a large part of the European countries.

To summarize, based on an in-depth qualitative analysis of how EM personnel in three settings (SOST, MH, and CH) experience stress-full interventions, we want to plea for a systemic approach to preserve mental health. In 2005, a panning article (109) strongly advised to implementation of multilevel Human Factor approaches in the medical sector. The results of the current study appear to be an echo of what may not always be heard by stakeholders. Therefore, we aimed at integrating the most acute shortages identified by the interviewees with existing literature to show how different components as presented in the SHELL model are interrelated. Therefore, training on stress- and risk awareness should be approached both on an individual and systemic level, knowing that there is clearly no “one-size-fits-all” manner. Furthermore, to cope with stress exposure, EM personnel is continuously searching to position themselves on a continuum between emotional disconnection from the patient to preserve operationality on the one hand and remaining enough empathic to preserve humanity on the other hand. Their excessive passion for the job may become unbalanced with the sacrifice of their own personal life (e.g., stress exposure, growing volatile and dangerous working environment, and family life), accepting that the exception becomes the rule. Therefore, when a lack of recognition from different layers of the system is present, the risk for moral injury and compassion fatigue is never far away, being a symptom of an unhealthy system rather than an unhealthy individual. Knowing that the interviews were taken before the COVID-19 pandemic, this risk might even be increased (110). Finally, we observed an attitude bias regarding the risk for skill decay, probably shaped by culture, again emphasizing the need for a systemic lens. When the needed attitudes and regulations, crucial for safety and mental health, are not implemented as an obvious component through the different layers of the entire system, one cannot expect them to become part of the daily behavioral alphabet. You can only perceive what you conceive.

## Data availability statement

The datasets presented in this article are not readily available in accordance with the ethical procedures of ICH-GCP and GDPR, and due to the data's military classification.

## Ethics statement

The studies involving human participants were reviewed and approved by Brugmann University Hospital. The patients/participants provided their written informed consent to participate in this study.

## Author contributions

MV and NP: writing. MV, DG, and NP: analysis. JVH and FG: data-collection. NP, FG, FD, and JVB: expert knowledge. NP, JVB, and FG: design. MV, NP, and FD: manuscript redaction. NP: supervision. All authors contributed to the article and approved the submitted version.
