# Supplementary material for: Walk the line: a systemic perspective on stress experienced by emergency medical personnel by comparing military and civilian prehospital settings
Source: Front Public Health. 2023 Jun 27;11:1136090. doi: 10.3389/fpubh.2023.1136090 (PMC10335750; doi:10.3389/fpubh.2023.1136090)
Supplement: Supplementary file 1 [file Table_1.docx]

| **Supplementary Table 1**  ***Stress exposure, stress perception and stress resistance*** | | | |
| --- | --- | --- | --- |
|  | **Military Hospital** | **SOST** | **Civil Hospital** |
| **Stress Exposure, Stress Perception and Stress Resistance** | “I have known the big fires, I saw all kinds of events, I have experienced the attack on the University Leuven – site Woluwe 1988, several explosions … the stress, I have known it now for 30 years … patients severely burned … transporting burned people by helicopter … every time, these are real tragedies, because there is always the family as well.”  “I was at sea, I was in a helicopter that almost crashed, I was in it, there was the airplane that couldn’t turn back for a repatriation … sanitary missions, the airplane in Luxemburg …”  “Someone with a weapon before you, some guys with a knife under the throat … then you say ‘what am I doing? Do I go back?’”  “Someone bleeding to death, euhm, that sticks in one’s memory, that is what gives you a kick.” | “… this was horrible, sick making. Because of its extremity, I guess…  These are young guys … they arrive in pieces. They are so skinny … They don’t eat anything, and they stink like drifters … young people, they arrive, yeah, sometimes just with a bullet wound in the leg … but others they arrive in pieces, but the heart is still working … you must do something, but it is disgusting …  “… It was a bit exciting, and I survived so I enjoyed it a lot.”  “The last mission …, bombs were dropped 5 km from where we were, so that stays in the memory. Cause when you think, if that would have been a few kilometers more aside, we would have been hit.” | “For instance, a boy of 17, we were called for a stab of a knife. When we arrived, the boy had a knife in the abdomen, and he was already dead. We could not do anything, there was already rigor mortis … this was my 6th dead person in 6 weeks … a defenestration, a bullet wound, a stab of a knife, every night there was something … and always during night, outdoor where it was dark and gloomy. The patients needed to be lighted.”  “There have been pre-hospitalization situations that were very stressful and during which we were afraid for our lives. For instance, we find a patient who was out of his mind and went for a big knife in the kitchen. We left the place and the person instituted legal proceedings against us for not administering medical care.”  “Once, a person became very aggressive and I was in his visual field, and he rushed to me with a shatter of a bottle, and I saw the glance in his eyes and knew he wanted to get me … I had several situations like that that we had to call to ask for reinforcement from the police…” |
| **Most stressful events** | “Children for example, that is more sensitive than adults… or cot death… that child is little, it’s a baby. In fact, I feel often more pity for the mother, how she is reacting or with the family, because, yes, the one who is dead is dead, yeah…”  “I don’t know a lot of jobs where there are children dying in your arms…what is touching me is of course the child, but also everything around: the family, managing …”  “I had five cot deaths in my career, and I know them still all by name… it is beyond expectation, a baby who dies.” | “I mean… (sighs) … a child is a child; it is more complicated. Everybody will say that I guess. Independent from whether it is ambulance, firemen…”  “I had a lot of dead persons … you have to live with them… but there’s always the tragedy, there is a family that is hurt…you enter a house, you enter in the life of other people, though. I was in houses of very rich but also … of guys where it was misery… but the pain is there.” | “… there are the children … we have much more empathy for children than for a 95-yr old granny who already had a life.”  “Everything that touches children or big traumas, the big accidents with children, it’s more touching when you are a parent… or violence or abuse against children” |
