# Supplementary material for: Walk the line: a systemic perspective on stress experienced by emergency medical personnel by comparing military and civilian prehospital settings
Source: Front Public Health. 2023 Jun 27;11:1136090. doi: 10.3389/fpubh.2023.1136090 (PMC10335750; doi:10.3389/fpubh.2023.1136090)
Supplement: Supplementary file 2 [file Table_2.docx]

| **Supplementary Table 2**  ***Other stress sources*** | | | |
| --- | --- | --- | --- |
|  | **Military Hospital** | **SOST** | **Civil Hospital** |
| **The Specific Environment: To Work with What You Have** | “… ask me again tomorrow, the beeps are not working today. So, if I want to go to bed tonight and the beeps don’t work, I can’t hear it when it is ringing…”  “… if equipment doesn’t work! Technical problems...” | “We are not working in an operating room with laminar airflow or that sort of things, adaptation of air condensation, no, we are sitting just on a ship or in a houseroom. So, there is an entire setting that disappears...” | “Also, the situations are different. Here everything is prepared. The patient lies in his bed and voilà… on intervention, you are sometimes crawling one over the other on a space of 2 by 2 meters.” |
| **The Relationship Between the Organization and EM-Personnel: A Mutual Cycle of Trust** | “The frustrations I have, is not from the work, the team is doing great, but it is everything around, euh, budgets etc.”  “… you are one of the long-serving persons, you have to go from your service… on the one hand they ask you to invest and on the other hand…” | “Recognition … once back [home], my personal luggage arrived in Iraq… we ordered blood and we received the blood three days later from Belgium. But our suitcase … never arrived.”  “Another important third stress factor is the dates of departure-return … last year I was on the list to leave January-February. Finally, after that I called the staff myself … they told me ‘oh but this is canceled’ … I find this respectless… that’s why people leave…” | “We have algorithms. We know how to treat the patient, even a critical patient. But if you have a problem of space and workload, there exists no algorithm to manage that.”  “… it is not the danger that is the greatest source of stress … but what people are not willing to, is to be exposed to that degree of stress without adequate support. Like that policeman said: ‘I risk my life, but I will need to work until my 67^th^.’ So, a lack of recognition. According to me, that’s a gigantic source of stress in our profession.” |
| **Relationship between (non-human aspects of) the working environment and the individual** | “I think that no nurse that does his 8th shift, that he is as relaxed as with the first and only after 4-5 days home that will have decreased a bit…the work rhythm is extremely high.”  “Life hygiene, it’s true that we don’t eat well, but I am lucky that my organism is well constituted… but for others, I can imagine that they can get corpulent.” | “When there is heavy artillery firing just next to the house where you are… and then the firing that comes back… even when we were having a break, it was stressful because you are in a tent on the airport, house style but still a tent… airport day and night, the reactors, a noise unimaginable… it weighs…” | “It is the combination of hours and workload … there are always peaks, for example now winter, the last two days were awful … there was no space in the surrounding hospitals…” |
| **The Impact of The Job on Family and Vice Versa** | “… and if I did the night and it was a long night, she knows she must leave me alone … then, euh, I am not pleasant, I harp on. I will not be angry, but I nag … Is that stress? I think rather fatigue. Little children are the same. If they are tired, they aren’t pleasant either (laughs).” | “My wife helped me, she was my therapist, she was my [emotional] punching ball. I could smash and smash, euhm… and she stood there.”  “… and I realize more and more these people in my family, euhm, yeah, they’ve another life, other jobs, they certainly won’t understand, euhm, we have become strangers” | “And those with whom you are at home sense very well when something is wrong … you are more closed, more distant …”  “At home, I had my wife, but we are in a divorce now …” |
