# Supplementary material for: Walk the line: a systemic perspective on stress experienced by emergency medical personnel by comparing military and civilian prehospital settings
Source: Front Public Health. 2023 Jun 27;11:1136090. doi: 10.3389/fpubh.2023.1136090 (PMC10335750; doi:10.3389/fpubh.2023.1136090)
Supplement: Supplementary file 3 [file Table_3.docx]

| **Supplementary Table 3**  ***Coping mechanisms*** | | | |
| --- | --- | --- | --- |
|  | **Military Hospital** | **SOST** | **Civil Hospital** |
| **The balance between emotional distance and empathy** | “… I think on the intensive care you are much longer in contact with the patients, and you get to meet the entire network and all the stories… on the emergency I often do not see the family. We are already gone with our ambulance.” | “… but it is not a compatriot and thus you have more distance…” | “it has been a work because I didn’t want to put a distance to the point of getting rigid and cold towards the patient. Because, obviously, to take care of the patient and their family and doing this well, there is also a communicative and relational side. And I knew I had to find the balance between putting barriers to protect myself, yet still allowing to care of the patient in his globality. But I can’t explain how I did.”  “… I guess that it evolves by getting older and with increased experience cause I know that the way I look to my work now and the emotional distance I have now, is not the same as 15 years ago; 15 years ago, my emotional distance was much larger because it was much more threatening … now I find it less threatening and thus I don’t need that much distance anymore.” |
| **Avoidant coping style** | “It’s a little backpack, it goes in there, euh, and it goes in there and when I go home, the backpack remains at the door.”  “I can enjoy a good glass of wine, hanging in the sofa, watching a movie” | “… on deployment, you get on the plane, and you return to Belgium and in fact you are already switching back the button… you are resetting yourself.”  “… they were still alive when arriving, yeah, half an hour later they are all dead… you can’t save them all, can you?”  “We have seen so much, it’s not that we get dulled, but you get habituated” | “We did everything we could, and the day continues for us”  “When I come home, I need to sit down quietly for 1-2h, for sure, before going to be… even when it is 1 or 2 o’clock in the night, I must do that. Eventual watching tv, playing on the I-Pad.” |
| **Emotion-oriented coping style** | “… depending on the doctor that was there as well, we sometimes may call the hospital where we dropped the patient off to ask what get out of it, how is he doing, or how was the scanner, euhm.”  “… there is always someone, even if it is someone who is sitting in the dispatch, who will come to you when there was a heavy intervention or a deceased or so.” | “I have this trick with my son, when there is something, then I make contact with my son via the stars. ‘I see you tonight in certain figures’”  “If needed and I am in a group I will say, I don’t manage anymore, help, there is a problem, euhm, I will not keep it for me.” | “… if the patient dies … I see it as something constructive and what I could do better in the future... there are always yeah… I should have done that, more in a negative way. The patient died and it is my fault. No, the patient had a bad pathology, you did the best you could…”  “you also want to hear or know ‘I did the right thing’, that is important as well… you don’t always have this feedback’” |
| **Task-oriented coping style** | “I think if you’re really busy with a patient… I was once, euhm, for a heavy car accident… to sketch you an example. You are so busy with the patient… we had to take him out of the car and if they ask me what color the car had, you don’t know. You are focused on the patient, and you do your thing…” | “You stay professional, yeah, the purpose is to do your job, yeah. That’s maybe the most important … yeah, that’s a sort of coping, that you rely on your reflexes, and you don’t panic.”  “You must just know what you are going to do. These are very specifically chosen steps in an operation. You know ‘that needs to be done first and then that, then he needs that instrument’ and then it is about adapting to the situation and how it proceeds.” | “I don’t know how to explain how I do. I think, on the moment itself, we are taking care, in the moment of being there for the patient. It just goes naturally”  “Oh yes, you feel your heart beating faster, you have difficulties to keep clear … but once you have noticed, you can retake your scheme of treatment and the procedures that allow you to pick up the thread.” |
| **Macho Culture or John Wayne Syndrome** | “It is here, but when the door closes… euhm… the colleagues, they are all a bit different, the male colleagues, they are the grandfathers … they are all so tough and euhm they all want to show it they are, but they are all little bears inside. And when there is something, these people are standing there for you”  “No… and I can immediately recall some situations where people were showing openly their emotions. Euhm… it is on the emergency … there is a flashing light culture… yeah, certainly when you compare this with other services.”  “Yes, you have indeed this macho culture. But I think though you need it on such a service, if not, you will go under.” | “Yes, … euhm, maybe we do that. Nobody wants too much to admit they are impressed.”  “I think in the army, it is certainly a bit there. But I don’t think it is about showing a brave face. I think that everybody or a lot of people are macho now and then… you want to be the best … and is this macho or professionalism? … it is not about pretending… but if I don’t care, I won’t effectively care a shit, yeah”  “… with young people this is different now.”  “Honestly… euhm… no, that’s not true… I remember medical doctors saying ‘ok, this was hard!’” | “… it depends on … from person to person. You always have those with a flashing light on their head… there are cowboys yeah … but sooner or later, it, euhm … it may have great consequences”  ““when one is too rigid, too cold because you have to be stoic and you should show that nothing touches you, one will not be able to communicate with the patient.”  “It is not warm and fluffy… euhm …but it is different now compared to 20 years ago … here in my team there is a good atmosphere.” |
| **Team investment: another mutual cycle of trust** | “… on the intensive care or on a unit of neurosurgery, this still exists yeah. There you still have the doctor in the white coat who makes his tour of 30 patients, briefs to the principal nurse who briefs to the nurse, so there’s more distance but we, we come at location with 3 and you have to solve it with the 3 of you…”  “To know your people in the field is very important… this is said on many conferences. It is 50 percent of the success of your contingency plan, that you know your people, that you can rely on them.”  “I don’t like to have a conflict with a colleague or to have delivered bad work…”  “Treating one another without respect is something I really find awful. It just happened to me when I entered today” | “This is totally horizontal. You only have specialists, and they work together… there are no ranks… everyone must, because we are a small group, listen to everyone and everyone must execute their task. It is not that the surgeon will only operate …”  “You really must be part of the group and if you can’t, you’re sent away, yes look, that’s a pity but it is better for the army.  Because, to live three months and a half with someone fulltime, 24/24, that’s something. It’s not always easy. It’s not the first time, so you must learn a bit to … to manage… voilà.  “I found it more difficult when there were ruptures in the team. … there has to be trust.” | ”It’s typical for emergency. You are binomial with your doctor, you cooperate. It’s not because he’s a doctor that he must take all the decisions. It’s possible that you must take responsibility for a patient and that he’s gonna do something else or vice versa.”  “It makes us strong also. We can say to one another whatever we want… whether these are direct or indirect colleagues or nurses or doctors…”  “We have nurses that for example have been working already for 10 or 15 years, they’re more experienced than young medical doctors and sometimes they know more … We need to be a team.”  “From above, we don’t have a lot of recognition, but we have it among colleagues… this recognition is sometimes very important. On the emergency you don’t have that a lot from the chief, patient, family etc.” |
